# Retraction: CUL4B promotes bladder cancer metastasis and induces epithelial-to-mesenchymal transition by activating the Wnt/β-catenin signaling pathway

**DOI:** 10.18632/oncotarget.28407

**Published:** 2024-08-05

**Authors:** Xia-Wa Mao, Jia-Quan Xiao, Gang Xu, Zhong-Yi Li, Hui-Feng Wu, Yi Li, Yi-Chun Zheng, Nan Zhang

**Affiliations:** ^1^Department of Urology, The Second Affiliated Hospital of Zhejiang University School of Medicine, Hangzhou 310009, P.R. China

**This article has been retracted:** Instances of image duplication have been discovered in this paper. Specifically, the WIF-1 panel of Figure 7C contains an overlap with the SFRP1+miR-27a inhibitors image in Figure 7C of an earlier published paper [1]. Figure 2B also contains multiple western blot overlaps with Figure 2B of another earlier published paper [2]. Therefore, the Editorial decision has been made to retract this paper. All authors agreed with the decision.

Original article: Oncotarget. 2017; 8:77241–77253. 77241-77253. https://doi.org/10.18632/oncotarget.20455
